# Particulate Matter Air Pollution: Effects on the Cardiovascular System

**DOI:** 10.3389/fendo.2018.00680

**Published:** 2018-11-16

**Authors:** Robert B. Hamanaka, Gökhan M. Mutlu

**Affiliations:** Section of Pulmonary and Critical Care Medicine, Department of Medicine, The University of Chicago, Chicago, IL, United States

**Keywords:** particulate matter, cardiovascular, lung, macrophage, inflammation, interleukin-6, thrombosis, coagulation

## Abstract

Air pollution is a complex mixture of gaseous and particulate components, each of which has detrimental effects on human health. While the composition of air pollution varies greatly depending on the source, studies from across the world have consistently shown that air pollution is an important modifiable risk factor for significantly increased morbidity and mortality. Moreover, clinical studies have generally shown a greater impact of particulate matter (PM) air pollution on health than the gaseous components. PM has wide-ranging deleterious effects on human health, particularly on the cardiovascular system. Both acute and chronic exposure to PM air pollution is associated with increased risk of death from cardiovascular diseases including ischemic heart disease, heart failure, and ischemic/thrombotic stroke. Particulate matter has also been shown to be an important endocrine disrupter, contributing to the development of metabolic diseases such as obesity and diabetes mellitus, which themselves are risk factors for cardiovascular disease. While the epidemiological evidence for the deleterious effects of PM air pollution on health is increasingly accepted, newer studies are shedding light on the mechanisms by which PM exerts its toxic effects. A greater understanding of how PM exerts toxic effects on human health is required in order to prevent and minimize the deleterious health effects of this ubiquitous environmental hazard. Air pollution is a growing public health problem and mortality due to air pollution is expected to double by 2050. Here, we review the epidemiological evidence for the cardiovascular effects of PM exposure and discuss current understanding about the biological mechanisms, by which PM exerts toxic effects on cardiovascular system to induce cardiovascular disease.

## Introduction

Ambient air pollution is a growing global health problem estimated to contribute to as many as 3.1 million all-cause deaths per year (1–3). Exposure to air pollution is the largest environmental health risk and ranks ninth among modifiable disease risk factors, above other common factors such as low physical activity, high cholesterol, and drug use (2). Most of the excess deaths attributable to air pollution exposure are due to acute ischemic/thrombotic cardiovascular events. In addition to excess mortality, air pollution is associated with significant reductions in healthy life years and worker productivity (2, 4). Air pollution may also be an important endocrine disrupter, contributing to the development of metabolic diseases such as obesity and diabetes mellitus (5). While the developing world is most burdened by air pollution-associated health effects, the association between air pollution and mortality is still evident in developed countries where pollution levels are well below target standards (6, 7). The purpose of this article is (1) to introduce the reader to the major studies that have established the link between particulate matter (PM) air pollution and human cardiovascular and metabolic disease and (2) to discuss the mechanisms by which PM mediates its biologic effects. For systematic review of the connection between air pollution and human disease, we refer the reader to several recent systematic reviews and meta-analyses (8–14).

## Air pollution

Air pollution is a complex mixture of gaseous and particulate components, each of which has detrimental effects on cardiovascular and respiratory systems. The composition of air pollution varies greatly, depending on the source, emission rate, and sunlight and wind conditions. Gaseous components of air pollution include nitrogen dioxide (NO_2_), nitric oxide (NO), sulfur dioxide (SO_2_), ozone (O_3_), and carbon monoxide (CO) (2, 15, 16). Particulate matter (PM) components of air pollution consist of carbonaceous particles with associated adsorbed organic chemicals and reactive metals. Common components of PM include nitrates, sulfates, polycyclic aromatic hydrocarbons, endotoxin, and metals such as iron, copper, nickel, zinc, and vanadium (2, 15, 17). PM is subclassified according to particle size into (a) coarse (PM_10_, diameter <10μm), (b) fine (PM_2.5_, diameter <2.5μm), and (c) ultrafine (PM_0.1_, diameter <0.1μm). Coarse particles derive from numerous natural and industrial sources and generally do not penetrate beyond the upper bronchus. Fine and ultrafine particles are produced through the combustion of fossil fuels and represent a greater threat to health than coarse particles as they penetrate into the small airways and alveoli (16–19). While the organic and metal components of particles vary with location, levels of PM_2.5_ have consistently correlated with negative cardiovascular outcomes regardless of location (15).

## Epidemiological studies linking Pm exposure to morbidity and mortality in humans

The association between high levels of PM air pollution and adverse health outcomes has been known since the first half of the twentieth century. Smog incidents in Meuse Valley, Belgium (1930), Donora, Pennsylvania (1948), and London, UK (1952) acutely caused increased hospitalizations and deaths, particularly in the elderly and those with preexisting cardiac and respiratory diseases. An estimated 4,000 people died as a direct result of the London smog with 100,000 more suffering adverse health effects (20, 21). These incidents resulted in policy changes including the implementation of Clean Air Act in 1970 (22). The reduction in PM levels have led to gradual reduction in PM-associated morbidity and mortality; however, recent epidemiologic studies still consistently show a link between PM exposure and cardiopulmonary mortality.

### Short-term exposure studies

The increased deaths due to the smog in Meuse Valley, Donora, and London clearly suggested that acute exposure to air pollution is associated with adverse health outcomes. These classic cases of air pollution-induced mortality represent extreme examples, with the London smog reaching air PM concentrations of 4.5 mg/m^3^ (World Health Organization current safety guideline is 25 μg/m^3^) (21). A large number of short-term exposure studies have evaluated the associations between less extreme levels of air pollution and daily changes in mortality (15, 18). A recent meta-analysis of 110 peer-reviewed studies revealed that every 10 μg/cm^3^ increase in PM_2.5_ concentration was associated with a 1.04% (95% CI 0.52%-1.56%) increase in all-cause mortality (10). Hospitalizations and mortality due to cardiovascular and respiratory illnesses were positively correlated with increases in PM_2.5_ concentrations.

Several large, multi-city studies have been conducted in both North America and Europe, the largest being the NMMAPS (National Morbidity, Mortality, and Air Pollution Study) (23–25) and APHEA (Air Pollution and Health: A European Approach) (26, 27) studies. Findings from these studies were remarkably consistent and demonstrated that PM levels are significantly associated with daily all-cause, cardiovascular, and pulmonary mortality. Seasonal and regional variations existed in both studies possibly attributable to different sources of pollutants, meteorological conditions, and population differences. For example, the APHEA study found a stronger effect of PM on daily mortality in cities with a larger contribution of traffic emissions to total PM. This is in agreement with a recent study on triggers of myocardial infarction (MI) in which traffic exposure was found to be as significant of a trigger of MI as physical exertion and alcohol use (28). The NMMAPS study also found that the relationship between PM exposure and mortality was independent of gaseous co-pollutants, including NO_2_, CO, and SO_2_.

Studies carried out in Asia and the developing world have generally shown smaller effects on daily mortality due to PM than studies from the United States and Europe. A recent meta-analysis of 85 studies from 12 low- and middle-income countries showed a 0.47% (95% CI 0.34-0.61) increase for cardiovascular mortality and 0.57% (95% CI 0.28-0.86) increase for respiratory mortality for every 10 μg/cm^3^ increase in PM_2.5_ concentration (14). The cities covered by this analysis have mean PM_2.5_ levels ranging from 56 to 179 μg/cm^3^, which is significantly higher than the mean the PM_2.5_ levels in cities in the US and Europe. The reduced concentration-response relationship between PM_2.5_ levels and mortality in these countries is likely due to the higher baseline PM level seen in these countries. Indeed, current evidence suggests that the concentration-response relationship between PM_2.5_ levels and mortality is biphasic (29–33). A steep concentration-response function is observed at lower PM concentrations, while the curve flattens at higher concentrations. A recent study from Beijing, China found that while the slope of the concentration-response curve flattened at higher PM concentrations, there was no saturation for increased risk of ischemic heart disease mortality, even at PM concentrations as high as 500 μg/cm^3^ (33).

The biphasic relationship between PM concentration and adverse health outcomes means that the major health benefits from reducing PM levels will occur in countries with already cleaner air and that improvements in cardiovascular health will be more difficult to achieve in countries with higher levels of air pollution unless they can achieve a drastic improvement in PM concentrations. The results of the NMMAPS and APHEA studies suggest that there is no “safe” threshold under which increases in PM are not associated with increased deaths.

### Long-term exposure studies

In addition to studies on the acute effects of PM exposure, studies on the effect of chronic exposure to PM have revealed negative effects on long-term health outcomes. The first of these was the Harvard Six Cities study, which prospectively measured the effect of air pollution on mortality in a cohort of 8,111 adults while controlling for individual risk factors, including smoking, body mass index, occupational exposures, hypertension, and diabetes (34). The adjusted mortality rate ratio for the most polluted cities compared with the least polluted cities was 1.26 (95% CI 1.08-1.47). Air pollution, particularly PM_2.5_ and sulfates was positively associated with death from lung cancer and cardiopulmonary diseases.

A larger study, the ACS Cancer Prevention II study linked risk factor data for 552,138 adults with air pollution data and mortality statistics (35, 36). Both PM_2.5_ and SO_2_ were positively correlated with all-cause, lung cancer, and cardiopulmonary mortality and every 10 μg/cm^3^ increase in PM_2.5_ was associated with a 4, 6 and 8% increased risk of all-cause, cardiopulmonary, and lung cancer mortality, respectively. Coarse particles and gaseous co-pollutants other than SO_2_ were not significantly related to mortality.

A study on 22 European cohorts within the multicenter European Study of Cohorts for Air Pollution Effects (ESCAPE) found an increased hazard ratio for all-cause mortality of 1.07 (95% CI 1.02-1.13) per 5 μg/cm^3^ PM_2.5_ (37). Significant associations persisted even among participants exposed to PM_2.5_ levels below the European annual mean limit value of 25 μg/cm^3^.

Overall, the evidence from both short-term and long-term exposure studies demonstrates a consistent association between increased air pollution exposure and mortality. While the magnitude of this effect is small, the ubiquity of air pollution exposure makes it a significant source of early mortality. A global assessment of mortality attributable to several risk factors, including air pollution was carried out in the Global Burden of Diseases, Injuries, and Risk Factors Study 2015 (GBD 2015) (38). This study estimated that PM_2.5_ is the fifth-ranking mortality risk factor, leading to 4.2 million deaths and 103.1 million disability-adjusted life-years in 2015. The largest number of deaths attributable to air pollution occurred in China with an estimated 1.11 million deaths. These numbers are similar to the findings of a recent study from China that attributed 40.3% of deaths due to stroke, 26.8% of deaths due to ischemic heart disease, 23.9% of deaths due to lung cancer, and 18.7% of deaths due to chronic obstructive pulmonary disease (COPD) to PM_2.5_ exposure (39). According to the GBD 2015 study, these represent the 1st, 2nd, 4th, and 5th leading causes of death in China, respectively (12).

### Susceptibility to PM-induced morbidity and mortality

Enhanced risk of cardiovascular death from PM exposure has been linked to old age, low socioeconomic status, preexisting heart and lung disease, and smoking. The APHENA (Air Pollution and Health: A Combined European and North American Approach) study, which analyzed data from the NMMAPS and APHEA studies found that the elderly and unemployed are at higher risk for the deleterious health effects associated with short-term exposure to PM (40). The ACS study found that mortality from ischemic heart disease was positively correlated with chronic PM_2.5_ exposure among never smokers, former smokers, and current smokers (41). However, the risk for death due to arrhythmia, hearth failure, and cardiac arrest was not elevated by PM_2.5_ for never smokers, but significantly elevated for former and current smokers.

Studies have not shown a clear association between race and susceptibility to PM-induced health effects (42–44). However, air pollution in non-white neighborhoods tends to be higher than in majority-white areas, resulting in exposure disparities (45). Indeed, inter-city gradients of PM (i.e., gradients among communities within a city) are associated with larger negative health effects than the average PM measurements within a city (46, 47).

Finally, it has been suggested that women may be more susceptible than men to the PM-induced health effects. Particularly, robust risk estimates have been reported for studies that include only women. The Women's Health Initiative Observational Study found that every 10 μg/cm^3^ increase in PM_2.5_ was associated with a 76% increase in fatal cardiovascular events while the Nurses' Health Study found that every 10 μg/cm^3^ increase in PM_10_ was associated with a 43% increase fatal coronary heart disease (48, 49). More recent large studies have given conflicting results (42, 43). On a global scale, exposure disparities may play a role in increased risk for women as use of biomass fuels for cooking in sub-Saharan Africa and south Asia expose women to disproportionately high levels of indoor air pollution (50).

### Interventional studies

The implementation of the 1970 Clean Air Act and following amendments resulted in a progressive decline in PM_2.5_ levels in the United States. As would be expected from the concentration-response curve of PM_2.5_ vs. mortality, extended analysis of the NMMAPS and Harvard Six Cities Study, among others, have revealed that reductions in PM_2.5_ concentrations over time are associated with reductions in mortality risk (51–53). Pope et al. showed that a reduction of 10 μg/cm^3^ in PM_2.5_ levels increased the life expectancy by 0.61 ± 0.20 years (54). Similar reductions in mortality have been seen after policy changes regulating the use of diesel in Tokyo, Japan and coal in Dublin, Ireland (55, 56).

## Exposure to Pm and cardiovascular disease

Deaths due to air pollution exposure result primarily from cardiovascular causes, with stronger associations with adverse effects of PM compared with gaseous co-pollutants (2, 7, 15). Chronic and acute exposure to elevated PM_2.5_ levels is closely associated with elevated risks for ischemic heart disease, heart failure, and cerebrovascular disease. Air pollution exacerbates existing heart conditions and appears to have a role in disease development.

Both long- and short-term studies have associated PM_2.5_ exposure with increased risk of fatal and non-fatal ischemic heart disease (33, 41, 48, 57). Risk of myocardial infarction is also associated with PM_2.5_ exposure (28, 58). The ACS study found increased risk of heart failure with PM_2.5_ exposure, although to a lesser degree than the association with ischemic heart disease. Additional studies and meta-analyses have associated both chronic and acute PM_2.5_ exposure with heart failure (9, 41, 59, 60). Significant associations also exist between PM_2.5_ exposure and cerebrovascular disease (48, 61). Short-term studies have shown that elevations in pollution increase the risk of ischemic, but not hemorrhagic stroke (8, 62).

### Subclinical effects

Exposure to PM air pollution is also correlated with subclinical pathologies underlying cardiovascular disease. These include systemic inflammation and oxidative stress, atherosclerosis, thrombosis, endothelial dysfunction, hypertension, cardiac remodeling, and arrhythmia.

#### Inflammation, oxidative stress, and atherosclerosis

PM inhalation induces inflammatory responses both within the lung and systemically. Exposure of human volunteers to PM via inhalation for 2 h resulted in increased pulmonary neutrophil numbers (63). Circulating levels of C-reactive protein, fibrinogen, IL-1β (interleukin-1β), IL-6 (interleukin-6), GM-CSF (Granulocyte-Macrophage Colony Stimulating Factor), and TNF-α (Tumor Necrosis Factor-α) have been shown to correlate with environmental PM exposure levels (63–66).

PM exposure is also associated with systemic markers of oxidative stress, including atherogenic precursors such as oxidized lipids (67–70). Using carotid artery intima-media thickness as a surrogate for atherosclerotic progression, several studies, including the Multi-Ethnic Study of Atherosclerosis (MESA) have shown that intima-media thickness correlates positively with long-term exposure to PM (71–74). Other studies have shown that coronary artery calcification correlates with residence in a city center or near a major roadway (75, 76).

Studies in the atherosclerosis model apolipoprotein E (ApoE) knockout mice have shown that exposure to PM results in elevated levels of oxidized low-density lipoproteins, lipid peroxidation, and systemic oxidative stress. This is associated with increased atheroma burden, and increased plaque cellularity and lipid content (77–80).

#### Hypercoagulability and thrombosis

Exposure to PM has been shown to induce a prothrombotic state, which may play a role in its ability to cause arterial thrombotic (myocardial infarction, ischemic/thrombotic cerebrovascular events) and venous thrombotic events (deep venous thrombosis) (81, 82). Exposure to PM induces the production of fibrinogen, and other factors that play a role in hemostasis including Von Willebrand factor, sCD62P, and sCD40L (65, 83–85). In addition to prothrombotic pathways, antifibrinolytic pathways are also activated by PM exposure. Plasminogen Activator Inhibitor-1 (PAI1) has been shown to be upregulated by PM exposure and tissue Plasminogen Activator (t-PA) activity is inhibited (85–88). These findings correlate with previous reports of PM-associated increases in plasma viscosity, platelet activation, and *ex vivo* coagulation (89–92).

The 2008 Summer Olympics in Beijing, China offered a unique opportunity to study the effects of PM exposure on cardiovascular biomarkers. As government-mandated restrictions on industrial and vehicular emissions were enacted, particulate and gaseous pollutants decreased. In test subjects, this corresponded with decreases in circulating levels of sCD62P and Von Willebrand factor. When restrictions were eased after the games, levels of these factors increased to pre-Olympic levels (84).

#### Endothelial dysfunction, increased blood pressure, and cardiac remodeling

Both short- and long-term exposure to PM has been correlated with changes in vascular function. Controlled exposure to diesel exhaust or concentrated ambient particles leads to vascular dysfunction characterized by acute arterial vasoconstriction and inhibition of response to vasodilators (86, 93–96). The MESA study found that chronic exposure to PM_2.5_ correlated with decreased flow-mediated dilation of the brachial artery and retinal arteriolar narrowing (97, 98).

Several studies have reported associations between chronic PM exposure and development of hypertension (99, 100). Controlled-exposure studies using acute exposure of humans to concentrated ambient particles or diesel exhaust have demonstrated rapid increases in systolic blood pressure following exposure (101, 102). Exposure to PM has also been shown to increase the risk of gestational hypertension and pre-eclampsia (11, 103, 104).

Finally, traffic exposure has been associated with both left and right ventricular hypertrophy, suggesting that pollution-associated vasoconstriction and hypertension may exacerbate congestive heart failure (105, 106). Similar results have been found in mice. A 3-month exposure of mice to concentrated ambient particles exacerbates cardiac hypertrophy and fibrosis in response to angiotensin II infusion (107). A longer, 9-month exposure of mice to concentrated ambient particles was sufficient to result in increased ventricular size, systolic and diastolic dysfunction, and myocardial fibrosis (108).

#### Cardiac electrical changes and irregular heart rhythm

In patients with implantable cardioverter defibrillators, positive associations have been made between short-term increases in air pollution and incidence of cardiac arrhythmias including atrial fibrillation, ventricular fibrillation, and ventricular tachycardia (109–112). Exposure to air pollution is also associated with, increased heart rate, electric instability, ectopic beats, ST-segment depression, repolarization irregularities, and changes in heart-rate variability (65, 113–120).

The strongest correlations between arrhythmia and pollution exposure have been found when analysis was restricted to a subgroup of patients with frequent arrhythmias, suggesting that risk of arrhythmia is restricted to the most susceptible individuals (109). Similarly, a murine study found that wild-type mice did not exhibit arrhythmias after exposure to PM; however, significant arrhythmias were seen in mice engineered to exhibit cardiomyopathic changes that closely resemble congestive heart failure (121). In rats, greater effects of PM exposure on arrhythmogenesis were seen in animals previously injected with monocrotaline to induce pulmonary vascular inflammation and hypertension (122).

#### Metabolic syndrome and insulin resistance

Several clinical studies have linked PM with insulin resistance and type II diabetes mellitus (DM) suggesting PM as a modifiable risk factor for DM, an important risk factor for cardiovascular disease. Significant positive correlations between PM exposure and fasting insulin levels and insulin resistance have been found in both adults and children (123–125). A large study conducted using data from both the United States Centers for Disease Control and Prevention and the Environmental Protection Agency found that diabetes prevalence increases by 1% with each 10 μg/m^3^ PM_2.5_ (126). Another study of over 3,500 individuals in Germany revealed that each 1 μg/m^3^ of traffic-related PM_2.5_ was associated with a relative risk for type II DM of 1.36 (95% CI.97-1.89) after adjusting for variables including age, gender, BMI, and socioeconomic status (127). This effect size was similar to that obtained by comparing individuals living close to a major road with those that live farther than 200 meters from a major road (127). A recent meta-analysis suggests that the correlation of PM with DM is stronger in women (13). How PM-associated insulin resistance and type II DM may interact with other PM-associated health effects to affect cardiovascular system is a complex question. For example, diabetics have been shown to be more susceptible to PM-associated endothelial dysfunction (128).

Animal studies have confirmed the effect of PM exposure on insulin sensitivity. Mice genetically susceptible to type II DM, or mice fed high-fat diet and exposed to PM exhibit increased insulin resistance, glucose intolerance, elevated fasting glucose, and increased visceral adiposity when compared with mice exposed to filtered air (129–131). Interestingly, young mice exposed to PM beginning at 3 weeks of age developed homeostatic insulin resistance after 10 weeks of exposure without additional stress indicating a developmental window of susceptibility to the effects of PM (132).

## Biological mechanisms

**Figure 1:**
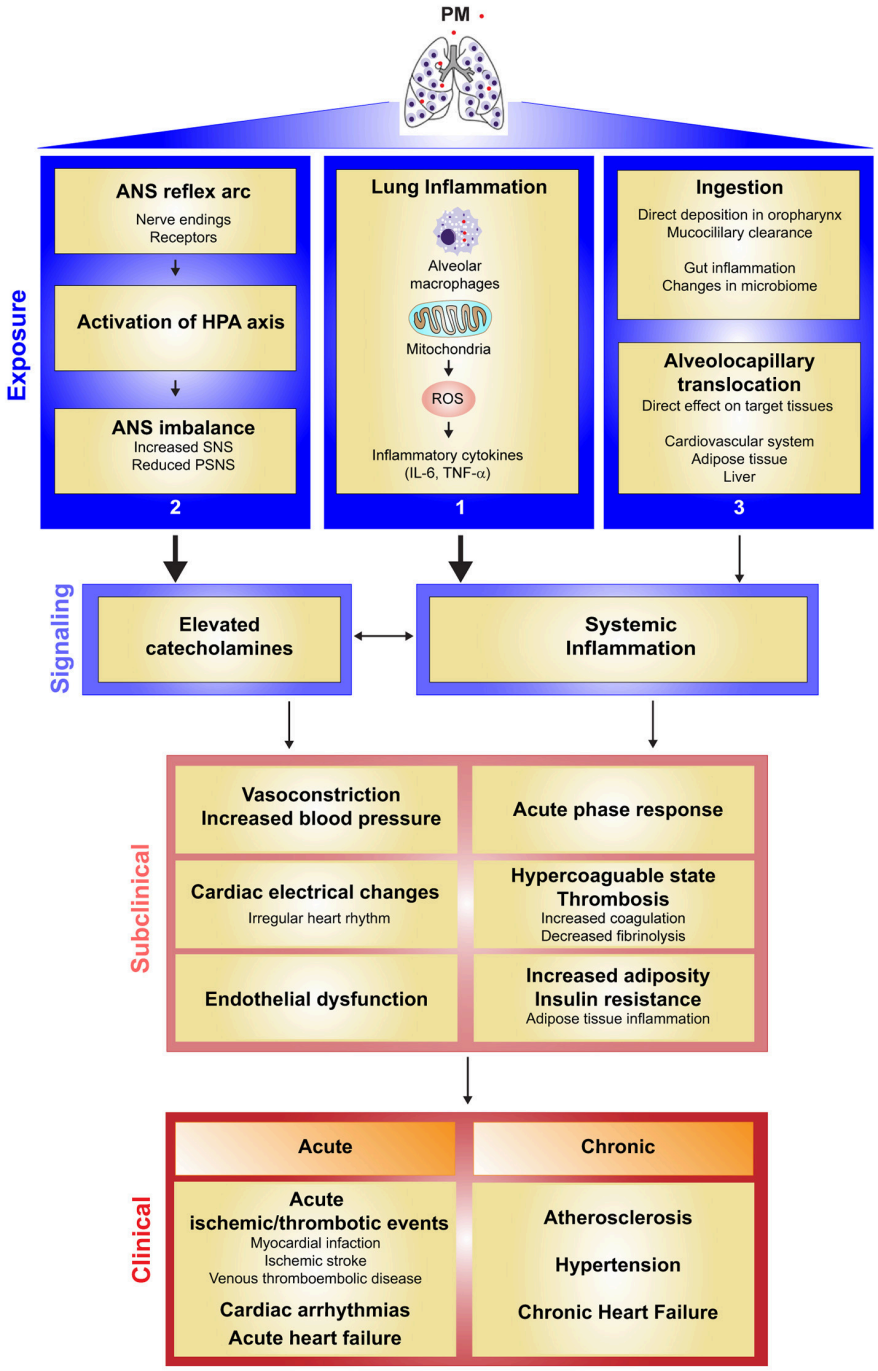
Current evidence for the mechanisms by which particulate matter air pollution causes cardiovascular health effects. **Exposure Level:** PM exposure is hypothesized to exert its effects on the cardiovascular system by three routes: (1) PM induces an inflammatory response in the lung. PM acts on the cells of the lung, including alveolar macrophages, leading to mitochondrial reactive oxygen species (mROS)-dependent pro-inflammatory cytokine production. (2) Inhaled PM acts on sensory receptors in the lung, promoting activation of the hypothalamic pituitary adrenal (HPA) axis and sympathetic pathway activation in the autonomic nervous system (ANS). (3) Other effects of PM exposure may be mediated by translocation of particles into the circulatory system, or by particle ingestion, which may promote inflammation in the gut. **Signaling Level:** Cytokines produced into the lung “spillover” into the circulation, leading to a systemic state of inflammation. Translocated particles as well as inflammation resulting from particle injection may also contribute to a general state of systemic inflammation. Sympathetic activation leads to elevated levels of circulating catecholamines. **Subclinical Level:** Systemic inflammation and elevated catecholamine levels act on target cells leading to acute phase response, hypercoagulable state (activation of coagulation, and suppressed fibrinolysis), vasoconstriction, increased blood pressure, cardiac electrical changes, endothelial dysfunction, and increased adiposity and insulin resistance complicated by adipose tissue inflammation. Elevated catecholamine levels due to ANS imbalance further increase inflammation. Sympathetic activation leads to increased catecholamine production, which increases heart rate and promotes vasoconstriction, endothelial dysfunction, and hypertension. **Clinical Level:** The combined effects of systemic inflammation and sympathetic activation on their cellular targets lead to the clinical effects of PM on cardiovascular disease. These effects are seen at both the acute level (acute ischemic/thrombotic events, cardiac arrhythmias, or acute heart failure), or at the chronic level (atherosclerosis, hypertension, and chronic heart failure).

Recent controlled exposure studies in both humans and animals have shed light on the biological mechanisms behind PM-induced cardiovascular disease (Figure 1). There are presently three hypotheses on the mechanisms by which PM exposure exerts its biological effect (7, 15, 16, 133). The first hypothesis proposes that PM inhalation activates inflammatory responses in the lung leading to a “spillover” effect and systemic inflammation, which promotes thrombosis, endothelial dysfunction, and atherosclerosis. The second hypothesis suggests that inhaled PM activates sensory receptors in the lung, leading to imbalance of the autonomic nervous system (ANS), favoring sympathetic pathways and leading to alterations in heart rate, vasoconstriction, endothelial dysfunction, and hypertension. The third hypothesis proposes that some particles, particularly ultrafine particles (PM_0.1_) can enter the circulation from the lung and interact directly with target tissues; however, this mechanism remains controversial. Recent evidence suggests that majority of ultrafine particles are cleared from the lung in a similar manner as larger particles (i.e., alveolar macrophage-mediated clearance to the larynx) (134, 135). Nevertheless, soluble material adsorbed to the surface of inhaled particles may pass into the circulation. Further studies will be required to determine whether any portion of inhaled particles is translocated into the bloodstream and if so, whether these translocated particles contribute to PM-associated pathologies. We will discuss the evidence for inflammatory and ANS signaling as regulators of the biologic effects of PM exposure. It should be noted that these pathways are not mutually exclusive. In fact, there is significant evidence that while each plays an important role in mediating the cardiovascular effects of pollution exposure, they may also interact to drive the PM-induced health effects.

### Reactive oxygen species and mitochondria

It is widely accepted that PM exerts many of its biologic effects via the generation of reactive oxygen species (ROS) and induction of oxidative stress responses (19, 136). Exposure to air pollution is associated with systemic markers of oxidative stress (67, 69, 70). At the cellular level, many cell types have been shown to respond to *in vitro* PM exposure with elevations in cellular ROS levels and oxidative stress. This includes nasal, airway, and lung epithelial cells (137–141), macrophages (142–144), endothelial cells (145, 146), cardiomyocytes (147, 148), gastrointestinal epithelial cells (149), epidermal keratinocytes (150), and corneal epithelial cells (151). Moreover, elevated ROS levels are required for PM-induced biologic effects as antioxidant treatment or inhibition of oxidant production is sufficient to inhibit downstream pathways including proinflammatory cytokine production and induction of apoptosis (137, 138, 152–155).

While PM-adsorbed chemicals and metals are capable of generating free radicals inside cells, cells can respond to stimuli with generation of ROS as signaling molecules (156). Mitochondrial generation of ROS has been found to be an important signaling regulator of the cellular response to PM. Indeed exposure to PM has been found to alter mitochondrial morphology and function (142, 151, 157, 158). PM exposure leads to oxidation of redox probes specifically targeted to mitochondria (149, 159). Furthermore, cells genetically engineered to lack mitochondrial ROS production or cells treated with mitochondria-targeted antioxidants or respiratory chain inhibitors have inhibited responses to PM, strongly supporting the role of mitochondria-derived ROS in PM-induced biologic effects (138, 153, 159–161).

### Alveolar macrophages

Alveolar Macrophages (AMs) reside on the luminal epithelial surface of alveoli and are crucial for lung development, surfactant homeostasis, and immune surveillance (162). These cells also represent a critical signaling node for the effects of PM on target organs such as heart and vasculature. Treatment of AMs *in vitro* with PM elicits a transcriptional upregulation of inflammatory cytokines including TNFα, IL-1β, IL-6, IL-8 and GM-CSF (64, 163). Lung epithelial cells also respond to PM treatment *in vitro* and the interaction between AMs and epithelial cells may regulate their response to PM (164, 165); however, studies in animals suggest that the response of AMs to PM exposure is required and sufficient for downstream cardiovascular effects.

Elimination of AMs in mice using liposomal clodronate inhibits both pulmonary and systemic accumulation of IL-6 or TNFα protein after exposure to PM (166, 167). The prothrombotic and endothelial-activating effects of PM exposure were also inhibited by clodronate, suggesting that the pro-inflammatory responses initiated by AMs in the lung promote the systemic and cardiovascular effects of PM exposure (166, 167). The ability of AMs to influence systemic responses to PM is supported by studies on bone marrow activation in rabbits. Instillation of PM into the lungs of rabbits results in increased release of polymorphonuclear leukocytes from bone marrow, with elevated numbers of circulating band cells, a marker of bone marrow activation (168). Similar responses have been seen in humans (169). Instillation of supernatants from human AMs treated with PM *ex vivo* has a similar ability to activate rabbit bone marrow as instillation of PM suggesting that PM-induced inflammatory responses in AMs regulate the systemic effects of PM on target cells and organs (170).

### Pro-inflammatory cytokines

#### Interleukin 6

Initially identified as a regulator of T cell activation and B cell differentiation, IL-6 is a pleotropic cytokine with key roles in diverse biological processes such as immune responses, the acute phase response and inflammation, hematopoiesis, vascular function, lipid metabolism, and neuroendocrine regulation (171, 172).

IL-6 is a major regulator of the acute phase response and stimulates hepatocytes to synthesize acute phase proteins, particularly C-reactive protein and coagulation factors (173, 174). IL-6 has been shown to increase the expression of coagulation factors including fibrinogen, tissue factor, Factor VIII and von Willebrand factor, and decrease anticoagulants including protein S and antithrombin (174). Recent reports in IL-6-deficient mice have demonstrated the critical role of IL-6 in promoting thrombotic events downstream of PM exposure. Exposure of wild-type mice to PM led to accelerated blood clotting and vascular thrombosis after FeCl_3_ application (166, 175). This accelerated clotting was associated with increased platelet count, increased Factor VIII activity, increased plasma thrombin antithrombin complexes, increased lung tissue factor levels, reduced prothrombin time, and reduced activated partial thromboplastin time. In IL-6 deficient mice, no increase in clotting capability was seen after exposure to PM, demonstrating the key role that IL-6 plays in regulating the prothrombotic effects of PM exposure.

Elevations in IL-6 may also promote vascular dysfunction after PM exposure. Administration of IL-6 to mice promotes endothelial dysfunction and impaired endothelium-dependent vasodilation (176). As in humans, exposure of mice to PM impairs vasodilation in response to acetylcholine. No significant change in vascular response was noted in IL-6 deficient mice after exposure to PM (177). Interestingly, in this same report, the authors demonstrated that instillation of recombinant IL-6 into the lungs of IL-6 knockout mice resulted in systemic elevations in IL-6 after endotoxin-mediated lung injury (177). While endotoxin is a stronger inducer of lung injury than PM, this finding provides support for the hypothesis that inflammatory cytokine induction in the lung can spillover into the circulation to promote systemic effects.

#### Tumor necrosis factor-α

TNFα is a pleiotropic cytokine originally identified as an endotoxin-inducible molecule with anti-cancer activity (178). TNFα has since been shown to be a critical regulator of the cytokine cascade in many inflammatory diseases and is a therapeutic target for a number of chronic inflammatory diseases (179, 180). Similar to IL-6, TNFα expression is induced in both AMs and lung epithelial cells after exposure to PM (64, 163, 165). TNFα also accumulates in both the lung and systemically after exposure of mice to PM (167, 175, 181, 182). Elimination of AMs with liposomal clodronate inhibited the accumulation of TNFα in plasma after exposure of mice to PM.

Surprisingly, studies in TNFα-deficient mice demonstrate that PM-mediated recruitment of neutrophils to the lung, as well as induction of cytokines, including IL-6, IL-8, and MCP-1 (monocyte chemoattractant protein 1), are independent of TNFα. (183, 184). This is despite the fact that TNFα is a known regulator of IL-6 expression (185, 186). TNFα was shown to be required for accumulation of PAI1 after exposure of mice to PM; however, TNFα remained dispensable for PM-mediated pro-thrombotic effects (175). More recent studies have shown that pulmonary T-cell recruitment is impaired after PM exposure in TNFα deficient mice (182) and that endothelial activation and impaired cardiac contractile function after PM exposure can be rescued by treatment with a TNFα-neutralizing antibody (167, 181).

### Sympathetic activation and endogenous catecholamines

The effects of PM exposure on heart rate variability and blood pressure indicate that air pollution may regulate the balance between the sympathetic and parasympathetic arms of the autonomic nervous system. Indeed, a study of Brazilian sugarcane harvesters found that during harvest time, when ambient PM is high due to sugarcane burning, workers' blood pressure and heart rate variability measurements correlated significantly with sympathetic nerve activity measured by microneurography (187). A more recent study found that elevated exposure to PM was associated with increased serum levels of norepinephrine and epinephrine, among other stress hormones (188).

Increased catecholamine levels have also been found in mice exposed to PM and this sympathetic activation has been shown to augment the inflammatory response and prothrombotic effects downstream of PM exposure (153). Deletion of β-adrenergic receptors either globally or in AMs alone resulted in reduced IL-6 accumulation after PM exposure. Inhibition of β-adrenergic receptors either genetically or pharmacologically prevented the prothrombotic effects of PM exposure while treatment of mice with the β-agonist formoterol further increased IL-6 accumulation and thrombosis after PM exposure (153). These findings from an experimental study have recently been confirmed in humans (188). Collectively, these results suggest that the PM-induced inflammation and modulation of the autonomic nervous system both contribute to the prothrombotic effects of PM exposure.

### Increased adiposity and adipose inflammation

Animal studies have shown that long-term PM exposure leads to increased adipocyte size and increased visceral fat mass (129, 189). PM exposure induced genes associated with lipogenesis in adipose tissue, impaired adipose mitochondrial function, and led to changes in circulating levels of leptin and adiponectin (130, 189–191). This increased adiposity was also associated with associated with increased macrophage infiltration into adipose tissue and induction of pro-inflammatory programs (129, 189).

Adipose inflammation is linked with insulin resistance (192). Indeed mice deficient for the NADPH oxidase subunit p47phox exhibited improved adipose inflammation and insulin resistance in response to PM exposure (132). Similar findings were found in mice deficient for the chemokine receptor CCR2 (131)

In humans, living near a major roadway (<60 m) is associated a 0.37 kg/m^2^ (95% CI: 0.10 to 0.65 kg/m^2^) increase in body mass index (BMI) when compared with those who live over 440 m away from a major road (193). The finding that inflammation is associated with PM-induced insulin resistance in mice is consistent with the findings of the SALIA (Study on the Influence of Air Pollution on Lung Inflammation and Aging), which demonstrated that Complement C3c, a marker of subclinical inflammation, is associated with PM exposure in a cohort of non-diabetic women. Elevated C3c was a strong independent predictor of diabetes development (194).

Animal studies have confirmed the effect of PM exposure on insulin sensitivity. Mice genetically susceptible to type II DM, or mice fed high-fat diet and exposed to PM exhibit increased insulin resistance, glucose intolerance, elevated fasting glucose, and increased visceral adiposity when compared with mice exposed to filtered air (129–131). Interestingly, young mice exposed to PM beginning at 3 weeks of age developed homeostatic insulin resistance after 10 weeks of exposure without additional stress indicating a developmental window of susceptibility to the effects of PM (132).

### Epigenetic changes

How the effects of air pollution exposure may endure after exposure is not clear; however studies from mice suggest that exposure early in life can have long lasting effects. Exposure of pregnant mice to diesel exhaust resulted in an increased susceptibility to pressure overload-induced heart failure in pups raised to adulthood (195). While the mechanisms behind this susceptibility is unknown, a potential mechanism for long-term disease susceptibility may lie in epigenetic changes that occur during exposure.

Epigenetic regulation of gene expression can result in transient, and potentially permanent changes in tissue function. Although studies are limited, air pollution exposure has been shown to affect multiple epigenetic mechanisms, including alterations in DNA methylation and histone modifications. Hypermethylation was observed in the DNA from sperm collected from mice exposed to particulate air pollution for 10 weeks when compared with mice exposed to filtered air (196). These changes were still evident when mice were examined 6 weeks after termination of exposure. In humans, hypomethylation in DNA repetitive elements has been seen in circulating leukocytes after exposure to particles (197, 198). Furthermore hypomethylation of LINE-1 elements correlates with increased risk for ischemic heart disease, stroke, and all-cause mortality (199).

Epigenetic regulation of certain genes by PM has been seen in cultured lung epithelial cells (200, 201); however, a genome-wide assessment of epigenetic changes induced in various tissues by PM exposure is yet to be carried out. A new large-scale study, sponsored by the National Institute of Environmental Health Sciences seeks to determine the genome-wide epigenetic effects of exposure to various environmental pollutants, including PM, on multiple tissues (202). The data collected by the TaRGET II (Toxicant Exposures and Responses by Genomic and Epigenomic Regulators of Transcription) Consortium will greatly advance the knowledge of the effects of air pollution exposure on the epigenome.

## Conclusions and future directions

There is abundant evidence that air pollution is a major contributor to cardiovascular morbidity and mortality. Exposure to pollution is a major modifiable risk factor in the prevention and management of cardiovascular disease; however, the health effects of air pollution are not limited to the cardiovascular system. PM also appears to be an important contributor to development of metabolic diseases including obesity and type II diabetes. Emerging evidence suggests that PM exposure affects timing of puberty and reproductive health in both men and women (203–208). Furthermore, air pollution exposure may affect other systems including the central nervous system as well as the gastrointestinal tract and microbiome. (149, 209, 210). At current projections, premature mortality due to air pollution exposure is expected to double by 2050 (1). Reducing the effect of air pollution on public health will require both policy efforts to reduce production of air pollution as well individual efforts to limit exposure, particularly for those with preexisting susceptibility to cardiovascular disease.

The effects of PM exposure on catecholamine levels, insulin resistance, adiposity, and reproductive health, suggest that PM exposure is an important endocrine disruptor. Mechanistically, little is known about how PM exposure affects the endocrine system. Endocrine disrupting compounds are found in both the gaseous and particulate components of air pollution (211–214), however, further research will be required to determine if these compounds are the specific causes for the adverse health outcomes associated with PM exposure.

The totality of the evidence suggests that there is no “safe” level of PM exposure. Therefore, in addition to efforts to reduce PM production and exposure, future studies should increasingly focus on mechanistic investigations to better understand how PM causes adverse health effects. Further exploration of the signaling mediators and epigenetic regulators of the effects of air pollution on health may lead to pharmacological agents capable of mitigating the detrimental effects of air pollution on health. This effort will require cell-based and animal studies utilizing real-life exposures to PM as well as translational research in humans.

Finally, there should be increased efforts at public education on the harmful effects of air pollution exposure, particularly by physicians with at risk patients. Air pollution exposure should be seen as a major modifiable risk factor for cardiovascular disease. The United States Environmental Protection Agency provides daily ozone and PM level readings for cities in the US, Canada, and Mexico. Greater dissemination of these readings may not only help those at risk for PM-related health effects, but also increase awareness of the impact of PM exposure on health, possibly increasing demand for policy changes to reduce air pollution production.

## Author contributions

RBH and GMM contributed equally and wrote the manuscript together.

### Conflict of interest statement

The authors declare that the research was conducted in the absence of any commercial or financial relationships that could be construed as a potential conflict of interest.
